# Genetic Control of Variegated *KIR* Gene Expression: Polymorphisms of the Bi-Directional *KIR3DL1* Promoter Are Associated with Distinct Frequencies of Gene Expression

**DOI:** 10.1371/journal.pgen.1000254

**Published:** 2008-11-14

**Authors:** Hongchuan Li, Véronique Pascal, Maureen P. Martin, Mary Carrington, Stephen K. Anderson

**Affiliations:** 1 Basic Research Program, SAIC-Frederick Inc., National Cancer Institute-Frederick, Frederick, Maryland, United States of America; 2 Cancer and Inflammation Program, Laboratory of Experimental Immunology, Center for Cancer Research, National Cancer Institute-Frederick, Frederick, Maryland, United States of America; The Jackson Laboratory, United States of America

## Abstract

Natural killer (NK) cells play an important role in the detection and elimination of tumors and virus-infected cells by the innate immune system. Human NK cells use cell surface receptors (KIR) for class I MHC to sense alterations of class I on potential target cells. Individual NK cells only express a subset of the available *KIR* genes, generating specialized NK cells that can specifically detect alteration of a particular class I molecule or group of molecules. The probabilistic behavior of human *KIR* bi-directional promoters is proposed to control the frequency of expression of these variegated genes. Analysis of a panel of donors has revealed the presence of several functionally relevant promoter polymorphisms clustered mainly in the inhibitory *KIR* family members, especially the *KIR3DL1* alleles. We demonstrate for the first time that promoter polymorphisms affecting the strength of competing sense and antisense promoters largely explain the differential frequency of expression of KIR3DL1 allotypes on NK cells. KIR3DL1/S1 subtypes have distinct biological activity and coding region variants of the *KIR3DL1/S1* gene strongly influence pathogenesis of HIV/AIDS and other human diseases. We propose that the polymorphisms shown in this study to regulate the frequency of KIR3DL1/S1 subtype expression on NK cells contribute substantially to the phenotypic variation across allotypes with respect to disease resistance.

## Author Summary

Natural killer (NK) cells represent a specialized blood cell that plays an important role in the detection of virus-infected or cancer cells. NK cells recognize and kill diseased cells using receptors for self antigens (HLA) that are frequently altered on aberrant cells. The HLA receptors are known as Killer cell Immunoglobulin-like Receptors, or KIR. Humans possess from four to 14 *KIR* receptor genes in their genome, and individual NK cells express a subset of the available *KIR* genes, generating specialized NK cells that detect alterations in specific HLA proteins. The mechanism of this unusual selective gene activation was recently shown by our group to be controlled by a probabilistic bi-directional promoter switch that turns on a given gene at a pre-determined frequency in the NK cell population. The current study shows that the properties of the switches in terms of the relative activity of forward (on) versus reverse (off) promoter activity is directly correlated with the frequency at which a given gene is expressed within the NK cell population. These results have important implications for our understanding of the role of NK cells in viral resistance and bone marrow transplants.

## Introduction

Natural killer (NK) cells play an important role in the detection and elimination of tumors and virus-infected cells by the innate immune system [1]. NK cells can identify stressed cells via cell surface receptors for class I MHC that sense alterations of these molecules on potential target cells [2]. Human NK cells express inhibitory receptors of the Killer cell Immunoglobulin-like Receptor family (KIR) that recognize HLA class I molecules [3],[4], whereas mouse NK cells use members of a lectin–related family (Ly49) to recognize mouse class I MHC [5]. Both gene families contain activating counterparts; however, the ligands of these activating receptors are not well characterized [6],[7]. Activating KIR lack the immunotyrosine inhibitory motif (ITIM) present in the intracellular domain of inhibitory KIR due to a carboxy-terminal truncation of the protein, and have thus been named as short forms of the receptors. For example, KIR3DS1 is an activating receptor highly related to the KIR3DL1 (long form) inhibitory receptor (http://www.ebi.ac.uk/ipd/kir/align.html).

**Figure 1:**
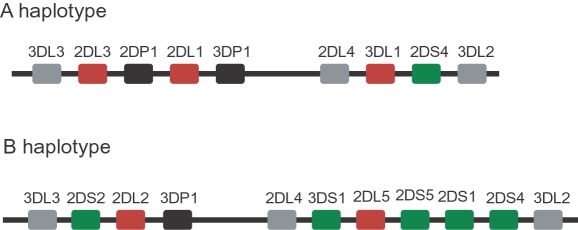
Organization of the *KIR* gene cluster. The *KIR* gene order is shown for the A haplotype and a representative B haplotype. Inhibitory KIR genes are shown as red boxes, whereas the activating genes are shown in green. The framework genes are indicated by grey boxes, and pseudogenes are indicated by black boxes. Each gene spans between 10–16 kb, and the intergenic distances are approximately 2 kb with the exception of the 14 kb region upstream of the *KIR2DL4* gene.

The *KIR* genes are located on chromosome 19 in a head to tail cluster with approximately 2 kb separating the polyadenylation signal of one gene from the translation initiation codon of the next. The number of genes present in *KIR* haplotypes is variable, however four genes (*KIR3DL3*, *KIR3DP1*, *KIR2DL4*, *KIR3DL2*) are present on virtually all haplotypes, and are thus considered as framework genes. Two major classes of *KIR* haplotypes have been identified. The A haplotype contains four genes in addition to the framework genes (*KIR2DL1*, *KIR2DL3*, *KIR3DL1*, *KIR2DS4*), representing a predominately inhibitory haplotype. There are many B haplotypes, containing various combinations of the activating *KIR* genes. The A haplotype and a representative B haplotype are shown in Figure 1.

Individual NK cells only express a subset of the available class I MHC receptors, presumably to generate specialized NK cells that can specifically detect alteration of a particular class I molecule or group of molecules [8]–[10]. The variegated expression of class I MHC receptors, KIR and Ly49, by NK cells is a unique case of selective transcriptional activation of a subset of genes present within a cluster. The B cell, T cell, and olfactory receptors are examples whereby a single receptor is selected from a large repertoire, and only one type of receptor is expressed per cell [11],[12]. In contrast, several *KIR* or *Ly49* genes can be expressed by a single NK cell in a stochastic manner [9],[10].

A considerable amount of information relating to the mechanisms controlling expression of the class I receptor genes has been acquired, and several general principles that apply to both the human and mouse systems have emerged. Expression is controlled by a stochastic mechanism; the probability of co-expression of two distinct inhibitory receptors is equal to the product of their individual frequencies, and NK cells appear to turn on class I MHC receptors until a self-reactive inhibitory receptor is present [13],[14]. Active receptor genes are hypo-methylated and silent genes are methylated [15]–[18]. Multiple promoters are present within each gene in both the *KIR* and *Ly49* clusters, including bi-directional promoters that are predicted to function as probabilistic switches controlling the probability of gene activation [19]–[22].

There is a high degree of polymorphism in the *KIR* gene family, including differences in haplotypic gene content among individuals [23]. Allelic variation has been observed for most *KIR* genes; however functional polymorphism within the promoter region of *KIR* genes has only been reported for *KIR2DL5* alleles, where loss of an AML-binding site was associated with the lack of *KIR2DL5* transcription [24]. Allelic variation in the *KIR3DL1* promoter has been reported [16]; however the functional consequences were not investigated.

A large number of *KIR3DL1* alleles have been identified, including an activating allele, *KIR3DS1*, making the *KIR3DL1/S1* locus unique within the cluster (http://www.ebi.ac.uk/ipd/kir/align.html). Numerous studies have demonstrated the effect of KIR3DL1 protein polymorphisms on the level of cell surface expression and the HLA recognition properties of the receptors [25]–[29]. There are currently at least four distinct categories of mean channel fluorescence intensity (MFI) of KIR3DL1 on the NK cell surface as detected by the DX9 and Z27 mAbs: low (KIR3DL1*028, *053), intermediate (KIR3DL1*005, *006, *007), high (KIR3DL1*001, *002, *003, *008, *015, *020) and null (KIR3DL1*004) [25]–[27],[30]. The distinct MFIs observed were attributed to differences in the level of cell surface expression rather than altered antibody-binding affinity. Although KIR3DL1 is known to bind multiple HLA allotypes that possess the Bw4 public serological epitope defined by residues 77–83 of the HLA α1 domain, the degree to which KIR3DL1 binds individual HLA-A and B alleles is variable among the different KIR3DL1 allotypes [28],[29]. Division of allelic groupings based on these differential characteristics of KIR3DL1/S1 molecules has associated strongly with HIV disease outcomes in genetic studies [31],[32].

The genetically-linked variability of the frequency of NK cells that express KIR3DL1 was established over 10 years ago [33], but the molecular basis of this variation has never been defined. The recent identification of bi-directional promoters in the *KIR* genes indicates that the relative strength of competing sense and antisense promoters may determine the probability of gene expression, similar to the model proposed for the control of *Ly49* gene expression by the Pro1 probabilistic switch [19],[20],[22]. To test this hypothesis, promoter polymorphisms that affect promoter activity were identified, and the frequency of receptor expression associated with individual alleles was determined. The availability of a monoclonal antibody (DX9) [34] that specifically recognizes KIR3DL1 and not KIR3DS1 provided the opportunity to specifically measure the frequency of expression of a single *KIR3DL1* allele in heterozygous *KIR3DL1/KIR3DS1* individuals and correlate the frequency of expression with specific promoter polymorphisms. The results reveal for the first time that specific *KIR3DL1* promoter polymorphisms affect the frequency of expression, which has consequences in terms of NK cell function in disease resistance.

## Results

### Identification of *KIR* Promoter Polymorphisms

**Figure 2:**
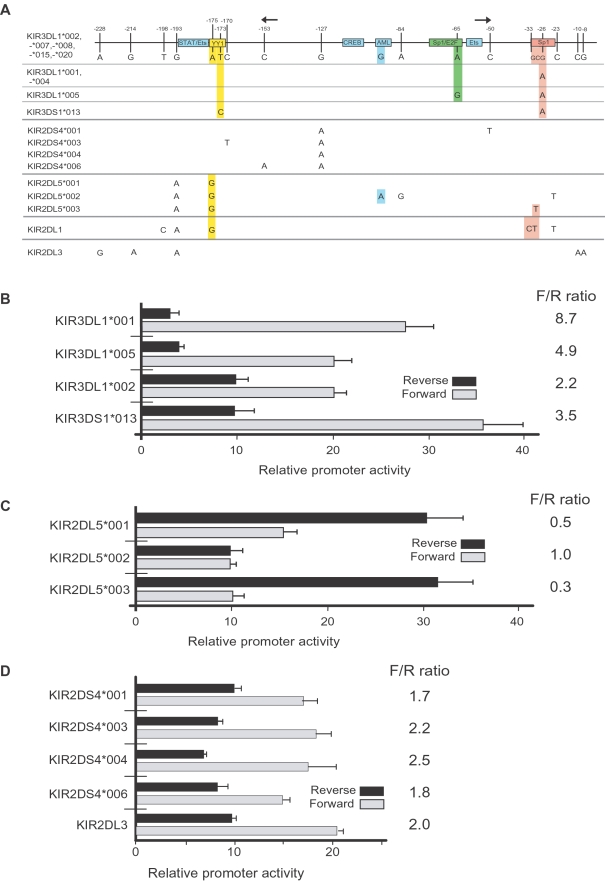
Impact of polymorphisms present in the *KIR3DL1*, *KIR2DS4*, *KIR2DL3*, and *KIR2DL5* genes on promoter activity. (A) A schematic of the *KIR* bi-directional promoter region is shown with the position of transcription factor binding sites indicated by labeled boxes. The sites of transcript initiation for the sense and antisense promoters are shown by the rightward and leftward arrows respectively. The vertical lines indicate the positions of polymorphic nucleotides relative to the KIR3DL1*002 allele. Numbers above the lines indicate the positions of the polymorphic residues relative to the start codon of the *KIR3DL1* gene. The nucleotide present at each variable position is shown for the KIR3DL1*002 gene and only differences are shown for the remaining alleles. (B) The promoter activities of *KIR3DL1/S1* promoter alleles are shown. The 229 bp core promoter region of the *KIR3DL1* alleles and *KIR3DS1* were cloned into the pGL3 reporter vector in the forward and reverse orientation, and the promoter activity of each construct was determined by transfection into the YT-Indy human NK cell line. The ratio of forward promoter activity to reverse activity (F/R ratio) is listed for each allele. (C) Promoter activities of *KIR2DL5* alleles. (D) Characterization of promoter activity of the *KIR2DS4* alleles and the *KIR2DL3* promoter. Values represent fold increase of luciferase activity relative to empty pGL3 vector. The mean and SD of at least 3 independent experiments are shown.

Three *KIR* genes were chosen for a detailed analysis of allelic variation in the promoter region based on the availability of specific antibodies to study their expression: *KIR3DL1* (detected by DX9 and Z27 mAbs); *KIR2DS4* (detected by FES172 mAb); *KIR2DL3* (detected by ECM41 mAb). Promoter polymorphisms were identified by sequencing PCR-generated clones of the core promoter region from individual donors, as well as analysis of KIR genomic sequences deposited in GenBank. Figure 2A shows the promoter polymorphisms observed in the donor population for the *KIR3DL1/S1* and *KIR2D*S4 genes as well as *KIR2DL5* polymorphisms identified in GenBank. The most frequently observed promoter sequence is shared by the KIR3DL1*002, -*007, -*008, -*015 and -*020 alleles (shown as the reference promoter sequence), and single nucleotide polymorphisms (SNPs) are shown for other *KIR3DL1* alleles as well as the *KIR2DS4* and *KIR2DL5* alleles. The *KIR2DL1* and *KIR2DL3* genes are shown as examples of *KIR* promoters that were not found to be polymorphic in the donor population. *KIR* genotyping identified 73 individuals in the NCI-Frederick donor population possessing at least one copy of the *KIR2DL3* gene; however, all of the *KIR2DL3* promoter sequences were identical. The KIR3DL1*004 promoter is identical to the KIR3DL1*001 promoter, but there may be no functional role for the KIR3DL1*004 allele, since the KIR protein produced by this allele is not expressed on the NK cell surface [26]. The *KIR3DL1/S1* alleles possess SNPs in the YY1, E2F, and Sp1 transcription factor binding sites, predicting functional differences in promoter activity of these alleles. On the other hand, the SNPs present in the *KIR2DS4* alleles and the *KIR2DL3* promoter are not associated with any predicted transcription factor binding sites, suggesting that the promoter alleles of these genes should have a similar level of activity.

### Distinct Bi-Directional Promoter Activity of Individual *KIR3DL1* Alleles

Previous studies have shown that the presence of ligand or competition from other KIR receptor-ligand pairs can influence the percentage of NK cells expressing a given KIR [14],[27],[35]. However, we have proposed that the primary determinant of the frequency of *KIR* gene activation is related to the probability of sense or antisense transcription from the proximal promoter [20],[22]. This model of probabilistic KIR expression predicts that there should be differences in the relative sense and antisense activities of individual *KIR* proximal promoter alleles to explain the observed differences in the percentage of NK cells that express different alleles of a given *KIR* gene. We previously observed that the KIR3DL1*001 and KIR3DL1*002 alleles have distinct bi-directional promoter characteristics [22]. To examine the effect of sequence differences observed in all of the *KIR3DL1/S1* promoter alleles, DNA fragments containing the previously identified core bi-directional promoter region (−229 to −1) [22] of the KIR3DL1*001, KIR3DL1*002, KIR3DL1*005, and *KIR3DS1* genes were cloned into the pGL3 vector in both orientations and the forward and reverse promoter activities were determined in transfected YT-Indy human NK cells. As shown in Figure 2B, the forward and reverse promoter activities of the individual *KIR3DL1* promoter alleles are distinct. Since the ratio of forward to reverse promoter activities should determine the probability of forward transcription and gene activation, the ratio is shown for each allele (Figure 2B). The KIR3DL1*001 promoter had the highest ratio of forward to reverse promoter activity and KIR3DL1*002 had the lowest, predicting that these two alleles should have the highest and lowest frequency of expression respectively. The transcriptional activities of the KIR3DL1*001 and *KIR3DS1* promoters in the forward direction are higher than those of the KIR3DL1*002 and KIR3DL1*005 promoters. The Sp1 site is disrupted (G→A) in the *KIR3DS1*, KIR3DL1*001 and KIR3DL1*005 alleles (Figure 2A); however, an increase in the strength of the forward promoter is not seen in the KIR3DL1*005 promoter, suggesting that the additional polymorphism within the E2F site at position −65 unique to the KIR3DL1*005 promoter (A→G) counteracts the positive effect of the SNP in the Sp1 site. A recent report has demonstrated that this polymorphism reduces E2F binding, resulting in reduced forward promoter activity [36]. The forward promoter activity of the *KIR3DS1* promoter was highest of the four tested, including that of the KIR3DL1*001 allele. There is an additional SNP in the YY1 site of the *KIR3DS1* promoter (T→C), and the YY1 site has been shown to inhibit forward and reverse promoter activities [22],[37]. Therefore, the increased forward and reverse activities of *KIR3DS1* relative to KIR3DL1*001 is likely due to a disruptive effect of the additional SNP in the YY1 site unique to the *KIR3DS1* promoter.

### Distinct Bi-Directional Promoter Activity of Individual *KIR2DL5* Alleles

Our previous characterization of the KIR2DL5*001 promoter indicated that disruption of the YY1 site resulted in a bidirectional element with dominant reverse promoter activity [22]. Examination of the KIR2DL5*003 promoter revealed the presence of an additional polymorphism in the Sp1 site. Figure 2C compares the promoter activity of KIR2DL5*003 to the *001 and *002 alleles. The disruption of the AML-binding site in the non-transcribed KIR2DL5*002 allele generates a promoter with weakened but balanced forward and reverse activity as previously shown for the *KIR3DP1* promoter [22]. The novel polymorphism in the Sp1 site of the KIR2DL5*003 promoter results in a further decrease in forward promoter activity. A recent report by Estafania et al. [38] has revealed that KIR2DL5 is expressed by only a small percentage of NK cells (∼5%), consistent with the dominant reverse promoter activity observed with the promoters of the two expressed alleles (*001 and *003). The reduced forward promoter activity of KIR2DL5*003 suggests that it will be expressed on an even lower percentage of NK cells than the KIR2DL5*001 gene.

### Lack of Functional Polymorphisms in the *KIR2DL3* and *KIR2DS4* Promoter Alleles

Although there were several promoter polymorphisms observed within the *KIR2DS4* genes, the SNPs observed were in regions lacking known transcription factor binding sites (Figure 2A). The promoter activities of the *KIR2DS4* alleles and the *KIR2DL3* gene were determined, and there are only small differences in the forward and reverse promoter activities of the *KIR2DS4* alleles and *KIR2DL3* as predicted by the lack of SNPs in the known transcription factor binding sites (Figure 2D).

### Allelic Variation in *KIR* Promoter Activity Is Controlled by Flanking YY1 and Sp1 Stes

With the exception of the non-transcribed KIR2DL5*002 allele that has an altered AML-binding site [24] (the only *KIR* allele known to have such a variant), the transcription factor binding sites within the core bi-directional *KIR* promoters are conserved between individual genes and alleles. Modulation of *KIR* bi-directional promoter activity appears to be due to polymorphisms in the YY1 and Sp1 sites that flank the core promoter region (Figure 2). The Sp1 transcription factor binding site is downstream of the major transcription start site of the *KIR* forward transcript and the YY1 binding site is downstream of the region where antisense transcription is initiated [22]. In vitro promoter assays demonstrated that disruption of the YY1 site is associated with increased promoter activity in the reverse orientation, whereas polymorphisms in the Sp1 site are associated with increased forward promoter activity [22]. These results indicate that Sp1 binding has an inhibitory effect on forward transcription whereas YY1 binding attenuates antisense transcription. The KIR3DL1*001 promoter has a SNP that disrupts the Sp1 site, possesses high forward transcriptional activity and low reverse activity. (Figure 2). The *KIR2DL1* promoter has 3 SNPs, one in the YY1 site, and two in the Sp1 site (Figure 2A). These changes result in a promoter with high transcriptional activity in both directions [22]. The KIR2DL5A*001 promoter has a single SNP that disrupts the YY1 site, leading to dominant reverse promoter activity. The additional Sp1 polymorphism present in the KIR2DL5*003 allele further suppresses forward transcriptional activity (Figure 2C). Taken together, these observations support a model where the probability of transcription in the sense or antisense direction is controlled by the flanking YY1 and Sp1 sites.

**Figure 3:**
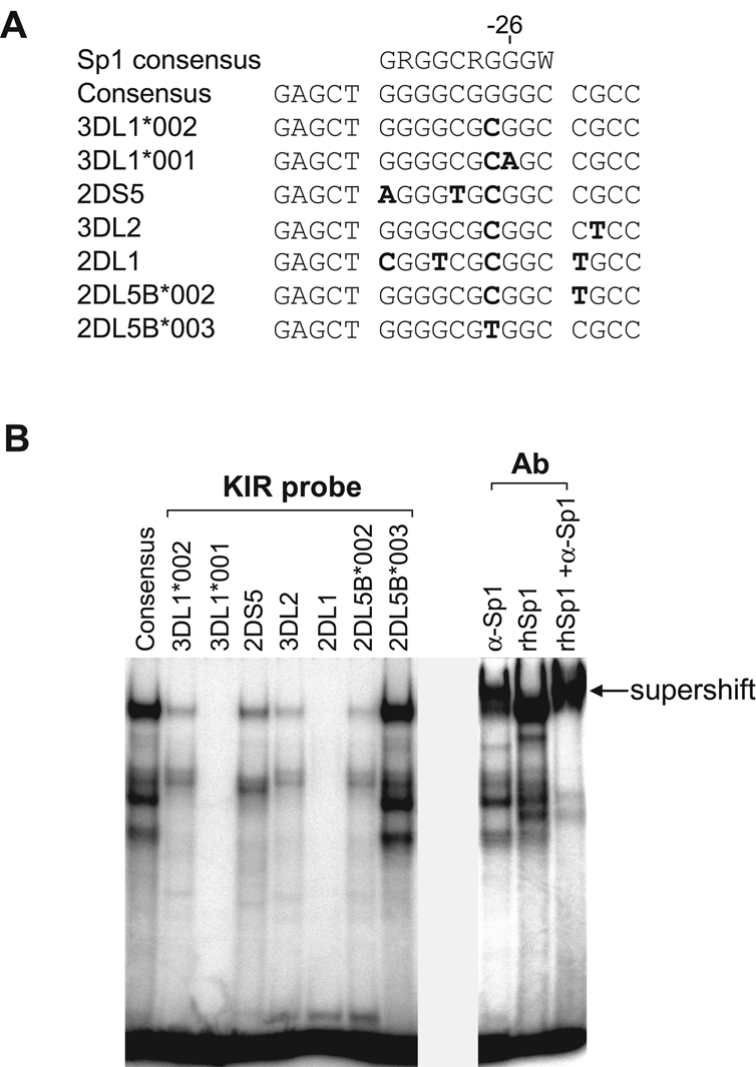
Effect of *KIR* promoter polymorphisms on Sp1 binding. EMSA analysis of the Sp1 binding region corresponding to the polymorphisms observed in *KIR* alleles. (A) Oligonucleotides used for EMSA. The sense strand of oligonucleotide probes corresponding to the predicted Sp1-binding region of the indicated *KIR* genes is shown. The nucleotide residue labeled (−26) corresponds to the same −26 position shown in Figure 2A. (B) EMSA analysis performed on YT nuclear extracts with probes indicated in A. The right panel shows a supershift of the Sp1 consensus probe from YT extracts or in the presence of recombinant human Sp1 protein (rhSp1).

Since the analysis of several *KIR* promoters revealed a significant effect of SNPs in the Sp1 site spanning nucleotides −24 to −33 relative to the start of translation, EMSA analysis of this region was performed with oligonucleotide probes containing the polymorphisms observed in the various promoter alleles as well as unique Sp1 site polymorphisms found in the *KIR2DS5* and *KIR3DL2* genes (Figure 3A). As shown in Figure 3B, polymorphisms associated with increased forward promoter activity (KIR3DL1*001 G→*A*; *KIR2DL1 G*→*T*) had reduced or undetectable Sp1 binding. The Sp1 site of the KIR2DL5*003 allele bound very strongly to Sp1, consistent with the decreased forward promoter activity of this allele. These results are consistent with the proposed modulation of forward promoter activity by Sp1 binding downstream of the major forward transcription initiation site.

### Quantitation of In Vivo *KIR3DL1* Antisense Transcript Levels

**Figure 4:**
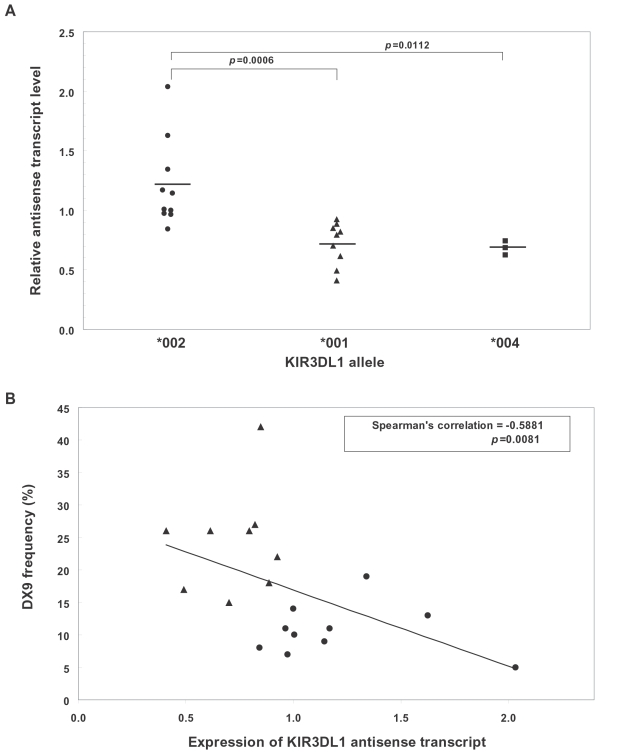
Quantitative analysis of *KIR3DL1/S1* antisense transcripts. (A) Comparison of relative antisense transcript levels in individuals bearing alleles shown to have high (*002) or low (*001/*004) antisense promoter activity *in vitro*. (B) Correlation of relative antisense transcript levels with frequency of NK cells expressing different KIR3DL1 allotypes. Symbols shown correspond to individuals possessing the *KIR* alleles as shown in A. The KIR3DL1*004 allele was removed from the analysis since it is not expressed on the NK cell surface.

In order to confirm the predicted effect of the observed changes in promoter activity associated with *KIR3DL1* promoter polymorphisms, the in vivo levels of antisense transcript were measured by quantitative PCR. Primers specific for *KIR3DL1* or *KIR3DS1* antisense transcripts were used, along with NKp46 coding region primers to control for the percentage of NK cells present in individual donor's blood. Figure 4A shows the result of antisense transcript quantitation in donors with *KIR3DL1* alleles that have either strong (KIR3DL1*002) or weak (KIR3DL1*001/*004) antisense promoter activity based on the transfection data shown in Figure 2B. There is a significant increase in the level of antisense transcript detected when an allele with a strong antisense promoter activity is present (KIR3DL1*002). The relationship between the frequency of receptor expression and antisense transcript levels was also studied (Figure 4B). A significant negative correlation was found between antisense level and the frequency of NK cells expressing a given allele, supporting the hypothesis that antisense transcription blocks *KIR* gene activation.

### Frequency of KIR Expression in Donors

**Figure 5:**
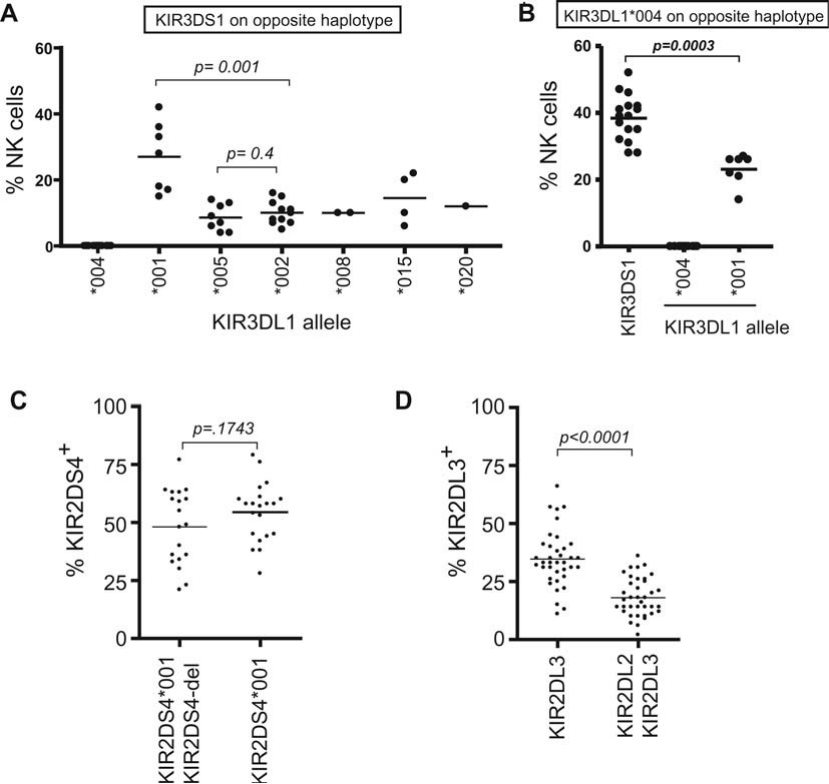
Frequency of expression of KIR3DL1/S1, KIR2DS4 and KIR2DL3 on the NK cell population. (A) The percentage of NK cells expressing KIR3DL1 in donors containing only one expressed inhibitory allele is indicated for individuals with a *KIR3DS1/3DL1* genotype. (B) The percentage of NK cells expressing either KIR3DS1 or the indicated KIR3DL1 allotypes was determined in individuals heterozygous for *KIR3DS1* or *KIR3DL1* and the KIR3DL1*004 allele (*004 is not expressed on the NK cell surface). (C) The percentage of donor peripheral blood NK cells expressing KIR2DS4 is shown. Individuals are grouped based on the presence of the expressed allele or the non-expressed *KIR2DS4* alleles that possess a 22 bp deletion (*KIR2DS4*-del) Heterozygous KIR2DS4*001/KIR2DS4-del donors possess one expressed *KIR2DS4* allele, whereas KIR2DS4*001 (in the absence of the null allele) individuals are expected to possess one copy of *KIR2DS4* but could potentially contain a second KIR2DS4*001 allele. (D) Expression of KIR2DL3 on peripheral blood NK cells is shown. Individuals typed as possessing *KIR2DL3* virtually always have two copies of the gene, whereas donors that have *KIR2DL2/2DL3* are expected to have only a single copy of *KIR2DL3*.

Although the percentage of NK cells that express individual *KIR* genes has been examined [27],[35], no evidence for allele-based differences in expression frequency based on promoter polymorphisms has ever been reported. In order to directly assess the frequency of expression of individual *KIR3DL1* alleles, donors possessing a single copy of the allele of interest were studied. The expression of a single *KIR3DL1* allele was examined with the DX9 antibody, which specifically reacts with KIR3DL1 and not KIR3DS1 in donors heterozygous for *KIR3DL1* and *KIR3DS1*. The Z27 monoclonal antibody, on the other hand, has weak reactivity with KIR3DS1 in addition to KIR3DL1 [29],[39],[40]; therefore, Z27 was used to measure the frequency of NK cells expressing KIR3DS1. Figure 5A shows the results obtained from an analysis of *KIR3DL1/KIR3DS1* heterozygous donors identified by KIR typing of individuals in the NCI-Frederick Research Donor Program. The KIR3DL1*001 allele is expressed by a significantly higher proportion of NK cells than any other *KIR3DL1* allele, consistent with the high level of forward transcriptional activity and low reverse activity observed for KIR3DL1*001 (F/R ratio of 8.7; Figure 2B). The frequency of expression of KIR3DS1 was analyzed in individuals that possessed the KIR3DL1*004 allele on the opposite haplotype (Figure 5B), since the KIR3DL1*004 allele is not expressed on the cell surface, thus avoiding the detection of KIR3DL1 by the Z27 mAb that reacts with both KIR3DL1 and KIR3DS1 [29],[39],[40]. A single *KIR3DS1* allele leads to the expression of KIR3DS1 on 30–50% of the NK cell population. This high frequency of expression was unexpected since the *KIR3DS1* promoter possesses an intermediate ratio of forward to reverse promoter activity (Figure 2B).

The lack of functional polymorphisms in the *KIR2DL3* and *KIR2DS4* gene promoters suggests that the corresponding KIR proteins should be expressed at similar frequencies on NK cell populations. An analysis of KIR2DL3 and KIR2DS4 expression using antibodies specific for each receptor showed that the frequency of expression observed for *KIR2DL3* (Figure 5D) is similar to that of KIR3DL1*002 (Figure 5A), consistent with the similar promoter characteristics of these two genes (Figure 2). Remarkably, the frequency of expression of KIR2DS4 was very high (mean = 48%; Figure 4C), even though the *KIR2DS4* promoter is also functionally equivalent to the KIR3DL1*002 promoter in the luciferase assay (Figure 2). This discrepancy may be due to a difference in the post-expression selection of NK cells expressing activating KIR. Along these same lines, it is also possible that the high frequency of KIR3DS1 expression (Figure 5B) may be due at least in part to post-expression selection.

### Effect of Gene Dosage on Expression Frequency

The comparison of KIR2DL3 expression in individuals possessing one copy of the *KIR2DL3* gene (*KIR2DL2/KIR2DL3* genotype) with individuals carrying two copies (*KIR2DL3/KIR2DL3* genotype) revealed a clear additive effect of gene dosage on expression frequency (Figure 5D). The mean KIR2DL3 expression frequency in individuals with one copy of the gene was 18%, whereas those individuals possessing two copies had a mean expression frequency of 35%. This result is consistent with the independent regulation of the two alleles. The expression frequency of two alleles should equal the sum of the frequency of expression of each allele minus the predicted frequency of cells expressing both alleles. In the case of *KIR2DL3*, since each allele should have the same probability of expression (*p*), the predicted expression of two alleles is 2*p*-*p* ^2^ (.36−.03) or 33%, in close agreement with the observed frequency of 35%. The effect of two copies of *KIR2DS4* on expression frequency could not be determined in this study since most donors possessing only the KIR2DS4*001 allele had a *KIR* B haplotype on the other chromosome and would not be expected to have two copies of KIR2DS4*001. Like the gene dosage effect of *KIR2DL3*, KIR3DS1 expression frequency also appears to be additive based on gene copy number. We had previously shown that individuals with two copies of *KIR3DS1* have a mean expression frequency of 61% [40] consistent with the expected frequency of 62% (.76−.14) predicted by the current observation that an individual *KIR3DS1* allele has a mean expression frequency of 38%.

## Discussion

There are undoubtedly many factors that contribute to the generation of the KIR repertoire, including the presence of ligands and competition between inhibitory receptors. Reports by Shilling *et al.* [33] and Yawata *et al.* [27] have shown clear effects of HLA on the frequency of expression; however, both studies concluded that the major factor controlling the degree of KIR expression was somehow related to the *KIR* genotype, but the mechanism was not resolved. The current study demonstrates for the first time that SNPs in transcription factor binding sites, which can occur amongst alleles of a single *KIR* gene, produce differences in the functional activity of the bi-directional *KIR* promoters that are associated with distinct frequencies of receptor expression.

A correlation between forward promoter activity and frequency of gene expression was observed for the bi-directional Pro1 promoter in the murine *Ly49* genes, since the reverse promoter activity was similar in all *Ly49* genes examined [19]. Forward transcription from the Pro1 promoter is required for activation of the downstream Pro2 promoter that is responsible for Ly49 expression in mature NK cells, since deletion of Pro1 abrogates Ly49 gene expression [41]. Although the probabilistic activation of KIR expression is also associated with the balance between sense and antisense transcription from a bi-directional promoter, the mechanism of gene activation must be distinct from the murine Ly49 system, since KIR expression in mature human NK cells originates from a bi-directional proximal promoter that appears to lose the ability to generate antisense transcripts in mature NK cells [22]. Perhaps antisense *KIR* transcription in developing human NK cells antagonizes the ability of sense transcripts from the upstream distal *KIR* promoter to open the locus, either by direct promoter competition or the production of double-stranded RNA in the *KIR* proximal promoter region.

The variation in sense versus antisense promoter activity of the *Ly49* probabilistic promoter is controlled by competition between overlapping C/EBP and TATA elements at either end of the bi-directional element. In contrast, the core bi-directional *KIR* promoter is conserved, and the variation in promoter strength between genes and alleles is controlled by flanking YY1 and Sp1 sites. The A→G substitution present in the upstream YY1 site of the *KIR2DL1* and *KIR2DL5* promoters has previously been shown to abrogate YY1 binding [37], consistent with the observed increase in reverse promoter activity associated with this SNP shown herein. An additional SNP in the YY1 site is present in the *KIR3DS1* gene, and this change is associated with an even higher level of forward promoter activity, but this is offset by an increased level of reverse activity as well. The frequency of KIR3DS1 expression was significantly higher than KIR3DL1*001; however, the analysis of the *KIR2DS4* gene suggests that additional factors beyond the characteristics of the *KIR* proximal promoter may control the frequency of NK cells expressing activating receptors. Although the in vitro transcriptional activities of the *KIR2DS4* promoter alleles are similar to the KIR3DL1*002 allele that is expressed on a low frequency of NK cells (∼10%), KIR2DS4 is expressed by 48% of NK cells on average. This discrepancy suggests that the KIR2DS4 and possibly KIR3DS1 subsets of NK cells undergo positive selection that increases the frequency of receptor expression in the NK pool. In this respect, it is worth noting that the mouse activating receptors Ly49D and Ly49H are co-expressed at a higher frequency than predicted by the “product rule”, suggesting that their expression is not governed by stochastic processes alone [42].

The current study provides the groundwork for further investigation of the role of promoter polymorphisms in *KIR* gene expression patterns. The identification and analysis of *KIR* promoter polymorphisms in more diverse donor populations together with the development of additional antibodies specific for individual *KIR* gene products will provide a more complete picture of the degree to which promoter polymorphisms modulate KIR expression frequency. Nevertheless, the information provided in this report is immediately applicable to studies of *KIR* locus variation on human disease and may explain some of the previous associations in this regard [43]. The quality of the NK response to a given pathogen is very likely to depend on the frequency of NK cells expressing the relevant KIR, which we have shown to be dependent on the specific promoter sequence driving transcription of the *KIR* allele/gene. KIR3DL1 allotypes that are expressed at a high level on the NK cell surface [25] associate with delayed progression to AIDS in HIV-infected individuals [31]. A high frequency of expression of these allotypes across the NK cell population would presumably lead to a larger population of mature, functional NK cells capable of detecting the loss of HLA-B. Indeed, KIR3DL1*001 and *KIR3DS1*, which show high levels of expression frequency are both protective against HIV-1, and *KIR3DS1* also shows protection against hepatitis C virus [44]. Theoretically, an improved NK sensing of HLA-loss through a greater number of NK cells expressing the appropriate sensors would enhance the ability of the individual to detect and eliminate virally infected cells that have decreased/altered HLA class I expression. Thus, it will be of much interest to determine the potential influence of these functionally-significant promoter variants on HIV disease as well as other human diseases.

## Materials and Methods

### Donors

Healthy volunteers were recruited through the NCI-Frederick Research Donor Program (http://www.ncifcrf.gov/rdp/). The *KIR* genotype of each donor was determined as previously described [45].

### Antibodies and Cell Lines

The monoclonal antibodies (mAb) used in this study were: PE-conjugated anti-CD158a/h (KIR2DL1/S1, EB6, IgG1); anti-CD158b1/b2/j (KIR2DL2/2DL3/2DS2, GL183, IgG1); anti-CD158e1/e2 (KIR3DL1/S1, ZIN276, IgG1); anti-CD158e1 (KIR3DL1, DX9, IgG1); anti-CD158i (KIR2DS4, FES172, IgG2a); FITC-conjugated anti-CD3 (IgG1, UCHT1); APC or PC5-conjugated anti-CD56 (IgG1, NKH.1) (Beckman Coulter Inc, Miami, FL); PE-Alexa Fluor 700-conjugated anti-CD3 (S4.1, IgG2a) (Invitrogen Caltag, Carlsbad, CA). The anti-CD158b2 (KIR2DL3, ECM41, IgM) [46] was kindly provided by Dr D. Mavilio, NIAID, Bethesda, USA. FITC-labeled goat IgG fraction to mouse IgM was purchased from MP Biomedical (Solon, OH). Appropriately labeled mouse isotype control mAb were purchased from the respective companies. YT-Indy cells were cultured in RPMI 1640 media containing 10% fetal bovine serum, L-Glutamine, and 100 U/ml each of penicillin and streptomycin.

### Flow Cytometric Analysis of KIR Expression on Peripheral Blood NK Cells

The proportion of NK cells expressing a particular KIR receptor was assessed in whole blood by three- or four-color flow cytometry. Briefly, 100 µl of EDTA-treated blood was incubated with the appropriate cocktail of mAbs. Erythrocytes were lysed with an ammonium chloride solution and the remaining cells were analyzed with a FACSort flow cytometer (Becton & Dickinson, San Jose, CA). Events (25,000) were collected in the lymphocyte gate and analyzed. NK cells were defined as CD3^−^CD56^+^ lymphocytes. KIR3DS1 expression on NK cells was investigated by using DX9 and ZIN276 (Z27) mAbs as previously described [37]. KIR2DL3 expression on NK cells was obtained by using GL183 and ECM41 mAbs. Results were expressed as percentages of NK cells positive for one given KIR receptor.

### Generation of Luciferase Reporter Plasmids

Promoter fragments were generated by PCR using a gene-specific forward primer starting at −229 and a reverse primer starting at −1 relative to the start codon of the gene. PCR products were cloned into the TOPO-TA vector (Invitrogen, Carlsbad, CA, USA), and inserts were excised with either *SacI/XhoI* or *XhoI/HindIII* and cloned into pGL3 (Promega, Madison, WI, USA) to generate constructs in both forward and reverse orientations. All constructs were verified by sequencing with specific primers. Sequence analysis was performed with the SeqWeb package at the NCI- Frederick supercomputing center.

### Cell Transfection and Luciferase Assays

YT-Indy cells were transfected by electroporation with a BTX ECM 830 (Gentronics, San Diego, CA, USA) set at 250 mV, with 3 pulses of 7 ms at an interval of 100 ms. A total of 5×10^6^ cells in 0.5 ml of serum free RPMI medium were transfected with 10 µg of the specific reporter construct plus 0.1 µg of the *Renilla* luciferase pRL-SV40 vector. Luciferase activity was assayed at 48 hr using the Dual-Luciferase Reporter Assay System (Promega) according to the manufacturer's instructions. The luciferase activity of the *KIR3DL1* promoter constructs was normalized relative to the activity of the *Renilla* luciferase produced by the pRL-SV40 control vector and each construct was tested in at least three independent experiments.

### Quantitation of *KIR3DL1* Antisense Transcripts

Total cellular RNA was isolated from peripheral blood mononuclear cells with the RNAqueous-4PCR Kit (Applied Biosystems/Ambion, Austin, TX), and further purified using DNase I according to the manufacturer's instructions. cDNA synthesis was carried out at 55°C using Oligo(dT)_18_ or *KIR3DL1/S1*-specific- TGGTTTATT(A)GTCACAATTG-3′ RT primers with the Transcriptor First Strand cDNA Synthesis Kit (Roche, Indianapolis, IN). Taqman real time RT-PCR primers and probes for target genes were: *KIR3DL1* antisense transcript Fwd- ATTGTCACAATTGCTCTGAAAACC -3′; Rev- CATGGCTTCCTGGAAATTGCT -3′ and probe: 5′(FAM)-CATGTTAGCACAGATTTTAGGCATCTCGTG -(MGB)3′. NKp46 Fwd- GGCTGTGTCTGAGTCAGAG -3′; Rev- GAGTTCATGTCCGGGATGTAG -3′ and probe 5′(VIC)- CATCTGGGCCGAGCCCCATTTCATG -(MGB)3′. The PCR reactions were performed in 20 µl final volume containing 30 ng of cDNA, 1×Master Mix (TaqMan Universal PCR Master Mix, ABI, CA), 500 nM of each primer and 100 nM of each probe, respectively. The thermal cycling conditions were 40 cycles of PCR amplification (UNG incubation: 50°C, 2 min; AmpliTaqGold activation: 95°C, 10 min; denaturation: 95°C, 15 s; annealing/extension: 60°C, 1 min) (7500 Fast Real-Time PCR System, Applied Biosystems, Foster City, CA). All assays were performed on the same plate in triplicate. Triplicate Ct values were analyzed using the comparative Ct (ΔΔCt) method as described by the manufacturer (Applied Biosystems, Foster City, CA, USA). The relative amount of *KIR3DL1* antisense transcript (2^−ΔΔCt^) was obtained by normalization to NKp46 and relative to the level of YT-Indy.

### Electrophoretic Mobility Shift Assays (EMSA)

Nuclear extracts were prepared from YT-Indy cells using the CellLytic NuCLEAR extraction kit (Sigma-Aldrich, St. Louis, MO). Protein concentration was measured with a Bio-Rad protein assay (Hercules, CA) and samples were stored at −70°C until use. All buffers contained a protease inhibitor cocktail (2 mM 4-(2-aminoethyl) benzenesulfonylfluoride, 1.4 pM *trans*-epoxysuccinyl-l-leucylamido [4-guanidinobutane], 130 pM bestatin, 1 µM leupeptin, and 0.3 pM aprotinin; Sigma-Aldrich). Eight double-stranded DNA oligonucleotide probes corresponding to the predicted Sp1-binding sequence of the *KIR* promoter alleles were synthesized (Figure 3A, sense strand shown). Sense and anti-sense oligonucleotides were annealed to generate the double-stranded oligonucleotides and labeled with [α-^32^P]dCTP (3000 Ci/mmol; Perkin Elmer, Waltham, MA) by fill-in using the Klenow fragment of DNA polymerase I (Invitrogen, Carlsbad, CA). Radio-labeled double-stranded oligonucleotides were purified using mini Quick Spin Oligo Columns (Roche, GmbH, Mannheim, Germany). DNA-protein binding reactions were performed in a 10 µl mixture containing 10 µg of nuclear protein and 1 µg of poly(dI-dC)poly(dI-dC) (Sigma-Aldrich) in 4% glycerol, 1 mM MgCl_2_, 0.5 mM EDTA, 0.5 mM DTT, 50 mM NaCl, 10 mM Tris-HCl (pH 7.5). After a 10-min incubation on ice, samples were incubated with 1 µl ^32^P-labeled oligonucleotide probe (20,000 cpm) at room temperature for 20 min, and then loaded on a 5% polyacrylamide gel (37∶5∶1). Electrophoresis was performed in 0.5×TBE buffer for 2 hours at 130 V and the gel was visualized by autoradiography after 2 days exposure at −70°C. For antibody supershift experiments, nuclear extracts were incubated with 2 µl of anti-Sp1 antibody (Santa Cruz Biotechnology, Santa Cruz, CA) for 1 h on ice prior to the addition of ^32^P- labeled DNA probe. After addition of labeled DNA-probe, the binding reaction was incubated for additional 20 min at room temperature. The human Sp1 recombinant protein (rhSP1, Promega, Madison, WI) was used as control. For competition analyses, unlabeled-competitor probe (Sp1 consensus) and AP2 probes were included in the binding reaction.

### Statistical Analysis

Statistical analysis of allele expression frequencies was performed using GraphPad Prism software. Comparison of distributions was performed using a Mann-Whitney *U* test. The correlations between the level of *KIR3DL1* antisense transcript and the frequency of NK cells expressing a given allele were assessed by Spearman's correlation coefficient. All p values reported were two-tailed, with significance defined as p<0.05.
